# Thermoelectric Properties of Novel Semimetals: A Case Study of YbMnSb_2_

**DOI:** 10.1002/adma.202003168

**Published:** 2020-12-09

**Authors:** Yu Pan, Feng‐Ren Fan, Xiaochen Hong, Bin He, Congcong Le, Walter Schnelle, Yangkun He, Kazuki Imasato, Horst Borrmann, Christian Hess, Bernd Büchner, Yan Sun, Chenguang Fu, G. Jeffrey Snyder, Claudia Felser

**Affiliations:** ^1^ Department of Solid State Chemistry Max Planck Institute for Chemical Physics of Solids Dresden 01187 Germany; ^2^ Leibniz‐Institute for Solid State and Materials Research (IFW‐Dresden) Helmholtzstraße 20 Dresden 01069 Germany; ^3^ Materials Science & Engineering (MSE) Northwestern University Evanston IL 60208 USA; ^4^ Institute for Solid‐State and Materials Physics Technical University Dresden Dresden 01062 Germany

**Keywords:** 2D Fermi surfaces, anisotropy, Dirac bands, Zintl compounds

## Abstract

The emerging class of topological materials provides a platform to engineer exotic electronic structures for a variety of applications. As complex band structures and Fermi surfaces can directly benefit thermoelectric performance it is important to identify the role of featured topological bands in thermoelectrics particularly when there are coexisting classic regular bands. In this work, the contribution of Dirac bands to thermoelectric performance and their ability to concurrently achieve large thermopower and low resistivity in novel semimetals is investigated. By examining the YbMnSb_2_ nodal line semimetal as an example, the Dirac bands appear to provide a low resistivity along the direction in which they are highly dispersive. Moreover, because of the regular‐band‐provided density of states, a large Seebeck coefficient over 160 µV K^−1^ at 300 K is achieved in both directions, which is very high for a semimetal with high carrier concentration. The combined highly dispersive Dirac and regular bands lead to ten times increase in power factor, reaching a value of 2.1 mW m^−1^ K^−2^ at 300 K. The present work highlights the potential of such novel semimetals for unusual electronic transport properties and guides strategies towards high thermoelectric performance.

## Graphical abstract

Single crystals of the YbMnSb_2_ nodal line semimetal demonstrate the synergetic contribution of Dirac bands and regular band to the thermoelectric performance. A high power factor over 2.1 mW m^−1^ K^−2^ is achieved due to both high mobility and large thermopower.

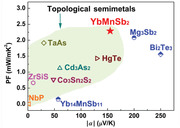

Band structure plays an essential role in determining the electrical transport properties of solids and thus lays the foundation of modern solid‐state electronic devices, for example, solar cells and thermoelectric modules. One of the most significant reasons sparking the development of thermoelectrics is the many new strategies for the engineering of the electronic structure, which can be dated back to late 1990s and continues to date.^[^ ^1^ ^]^ In the past decades, the distortion of the electronic density of states (DOS)^[^ ^2^ ^]^ and the convergence of electronic bands^[^ ^3^ ^]^ have significantly advanced the development of high‐performance thermoelectric semiconductors suggesting complex band structures and particularly complex Fermi surfaces benefit thermoelectric performance.^[^ ^4^ ^]^ In recent years, the discovery of topological materials has provided an abundance of exotic electronic structures, which provide new opportunities for exotic electronic structures that produce new functionalities such as unconventional thermoelectric performance.^[^ ^5^, ^6^, ^7^ ^]^

Since the first 3D topological insulators are discovered in Bi_2_Te_3_‐based alloys,^[^ ^8^ ^]^ the best room‐temperature thermoelectric materials up to now,^[^ ^9^, ^10^ ^]^ the relationship between topological insulators and thermoelectric materials is highly attractive. They are found to share some same characteristics, for example, composed of heavy elements and narrow band gap.^[^ ^11^, ^12^ ^]^ This is because the strong spin–orbit coupling (SOC) induced by the heavy elements opens the gap between the inverted bands, forming the topological states, and also produces the complicated bulk bands benefiting the thermoelectric performance.^[^ ^13^, ^14^ ^]^ On the other hand, heavy elements lead to a low thermal conductivity. However, the direct evidence for using the exotic surface states of topological insulators to enhance the thermoelectric performance, particularly for the 3D materials, has been rarely reported. The most important aspect of topological insulators lies in the usage of the topological surface states, or the edge states in 2D materials, to realize novel new physical effects, such as quantum spin Hall effect and quantum anomalous Hall effect.^[^ ^15^, ^16^ ^]^ In these cases, it is crucial to tune the Fermi level *E*_F_ into the forbidden gap so that the topological edge states can dominate the transport properties while keeping the bulk bands as insulating as possible. In contrast, good thermoelectric performance is generally obtained when the transport properties are dominated by the bulk electronic bands. Therefore, robust topological states in the bulk form have become new candidates in thermoelectric society.

The discoveries of novel topological semimetals with robust topological states, for instance, Dirac/Weyl semimetals, nodal line semimetals, have offered a new platform for the investigation of exotic transport properties.^[^ ^17^, ^18^ ^]^ However, the usual characteristic of “semimetals” of having small thermopower and high bipolar thermal conductivity due to the conduction of two types of charge carriers make these materials be less explored in conventional thermoelectrics. Nevertheless, it can be a different case in these novel semimetals if the degenerate bands are highly dispersive and are accompanied with a much larger regular band. This would lead to a situation in which the major charge carriers are much more than the minor charger carriers, making it possible to overcome the strong deterioration of thermoelectric performance from bipolar effect. Therefore, finding a novel semimetal without a strong bipolar effect is of great significance. It can not only shed light on the understanding of exotic electronic structures but also advances the exploration of such novel topological semimetals as exotic electronic materials as well as potential high‐performance thermoelectric materials. Even if such materials are not suitable for thermoelectric use, the study of Seebeck effect and conductivity experiments can give insight into exotic electron transport phenomena and identify new transport mechanisms.^[^ ^19^ ^]^

The difficulty in optimizing the thermoelectric performance lies in the intercoupling of the transport parameters, as denoted in the expression of the thermoelectric figure of merit *zT*, *zT* = (α^2^/*ρκ*)*T*, where ρ is the electrical resistivity, α is the Seebeck coefficient, κ is the thermal conductivity, and *T* is the absolute temperature.^[^ ^20^ ^]^ The coupling of these electrical transport parameters is deeply rooted in the intrinsic electronic structure. For example, the competition between α and ρ is best described by means of the electronic quality factor or weighted mobility μ_w_ which is the charge carrier mobility μ times the DOS effective mass *m* ^*^ (μ_w_ = (*m* ^*^/*m*_e_)^1.5^μ). Normally we think of the effective mass of a single band *m*_b_^*^, as inversely proportional to the curvature of electronic bands. At a constant charge carrier concentration, dispersive (light) bands with small *m*_b_^*^ are desired to achieve high mobility μ, while flat (heavy) band with large *m*_b_^*^ is thought to have a large |α|, as schematically shown in **Figure** **1**a. This suggests that by simply adopting an either small or large effective mass would hardly be satisfying for high thermoelectric performance.

**Figure 1:**
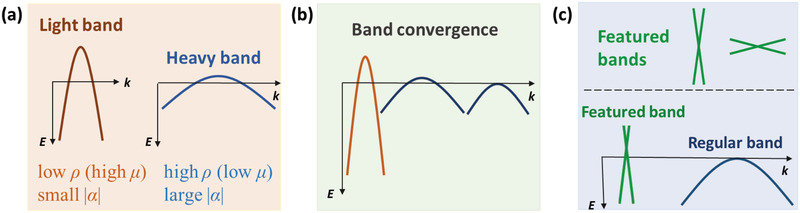
Schematic illustrations of band engineering strategies. a) At a constant charge carrier concentration, a light band with large curvature gives rise to high mobility μ (low resistivity ρ) and small thermopower |α|, while a heavy band with flatter dispersion leads to low μ (high ρ) and large |α|. b) Band convergence. c) Featured bands (bands that with Dirac/Weyl points or nodal ring in novel semimetals) with different degrees of dispersion (top), and a proposed band configuration of a highly dispersive featured band and a regular band in a novel semimetal (bottom).

To overcome this compromise, band engineering strategies have been put forward to improve the electrical performance of thermoelectric materials. As schematically illustrated in Figure 1b, one creative way is named as band convergence, in which more than one light band are aligned near the *E*_F_ leading to a higher band degeneracy. Each light band acts as a “high speed way” for charge carriers and altogether provide a large *m* ^*^ through improving the number of electronic valleys favoring a large α. This strategy has been widely implanted in many good thermoelectric materials, such as PbTe‐alloys,^[^ ^3^ ^]^ Bi_0.5_Sb_1.5_Te_3_,^[^ ^21^ ^]^ Mg_2_(SiSn),^[^ ^22^ ^]^ SnTe,^[^ ^23^, ^24^ ^]^ etc.^[^ ^25^ ^]^ A common feature for most of these successful systems is that they contain a light, high mobility band at lower energy than the multi‐valley bands that drives the thermoelectric performance. This may be due to the light band preventing localization of states in the heavy band.

Inspired by these band engineering strategies, we investigated the exotic thermoelectric transport properties of the novel semimetals which exhibit the coexistence of highly dispersive featured bands and classic regular bands. First, the highly dispersive featured bands can ensure a high charge carrier mobility. Second, the coexisting regular band which is usually much heavier than the highly dispersive topological band would provide a large DOS and thermopower. Herein it is worth noting that a linear band alone is unable to result in promising thermoelectric performance. Though linear bands can ensure excellent mobility, they have a very small contribution to thermopower. According to the Boltzmann transport description, α is derived from the velocity and DOS of the electrons in the band structure, which are both proportional to the slope of the band. Mathematically this is well described by the Mott model which is valid for bands that are differentiable near the Fermi level.^[^ ^26^ ^]^

(1) $$\begin{array}{c} \alpha^{M}\approx \frac{\pi^{2}}{3}\frac{k_{B}^{2}T}{e}{\left(\frac{2\partial v}{v\partial E}+\frac{\partial \tau }{\tau \partial E}+\frac{\partial g}{g\partial E}\right)\;|}_{E=E_{F}} \end{array}$$

where *v* is group velocity, *g* is DOS, τ is scattering time. For a linear band, *v* and *g* are constants and so the thermopower depends only on the scattering. Under energy independent scattering mechanisms, the linear aspect of a linear band has actually little contribution to thermopower, which is also demonstrated experimentally and with calculations of Skutterudite CoSb_3_.^[^ ^25^ ^]^ While it is worth noting that, for Dirac bands, if something unconventional (e.g., strong energy dependent scattering mechanism exists)^[^ ^27^ ^]^ happens, the thermopower can be larger than expected. Therefore, novel semimetals combining featured band and regular band would be an interesting platform to study the exotic thermoelectric transport properties, which can be promising for simultaneous achievements of high mobility and large thermopower.

Within the known novel semimetals, the combination of featured bands with regular bands is actually not uncommon. The interesting question arises as to which kind of configuration of the coexisting bands is most beneficial for thermoelectrics. In reality, the featured bands (bands with Dirac or Weyl points or nodal ring, etc.) can be highly dispersive, but can also be very flat (as shown in the upper panel in Figure 1c), and the relative positions between the featured bands and a regular band change from one compound to another. One desirable band structure of a novel semimetal for high thermoelectric performance is schematically illustrated in Figure 1c, where a highly dispersive band is coexisted with a regular band locating slightly below. This band configuration would not only benefit the simultaneous gain of μ and |α|, but also make the concentration of major charge carriers much more than the minor ones. Both the regular band and the featured band contribute to the major charge carrier, while the minor charge carrier only originates from the extremely light featured band. From this point of view, novel semimetal differs from conventional semimetals by avoiding the equal conduction of two types of charge carriers, making them possible to be promising thermoelectric materials.

In practical experiments, how to identify the contribution of featured bands to the transport properties is another key challenge. Here, we propose to investigate novel semimetals with strongly anisotropic 2D Dirac bands and nearly isotropic 3D regular bands and see if they unveil any unconventional properties. Because of the different anisotropy, 2D Dirac bands and 3D regular bands contribute differently to the electrical transport properties along varied crystal directions. Therefore, by measuring the anisotropic electrical transport properties, the contribution of Dirac bands to the electrical transport properties can be clearly evidenced.

YbMnSb_2_ has been found to be a novel semimetal with a coexistence of a regular hole pocket and 2D Dirac Fermi surfaces near the Fermi level.^[^ ^28^ ^]^ Most importantly, the 2D Dirac bands show strong anisotropy along different crystal directions while the 3D regular band has less anisotropy. Crystallized in a tetragonal lattice with a space group P4/nmm (**Figure** **2**a), first‐principle calculations (Figure 2b) denote a traditional regular hole pocket near Γ point (as shown by “T”) and Dirac bands along the Γ‐M, Γ‐X and Γ‐A directions (as marked by “D”). To understand the topological property of YbMnSb_2_, symmetry and SOC effect are considered, and can be compared to CaFeAs_2_, which has the similar crystal structure and has been well investigated. When with SOC, CaFeAs_2_ is a Z_2_ topological insulator due to band inversion at X point.^[^ ^29^ ^]^ However, since Sb1 atoms has no center‐shift behavior as As1, band inversion would happen at both X and Y points because of C_4_ rotational symmetry, making YbMnSb_2_ to be a topological trivial insulator. Nevertheless, when without SOC, there would be a nodal ring existing in the *k_z_* = 0 plane in YbMnSb_2_. Notably, even if the SOC is included, the open gap in the nodal ring is less than 10 meV in the DFT calculation, since the spin‐flip term 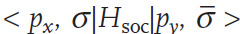 (in which *p_x_*, *p_y_* are the orbitals of Sb1, 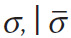 presents the spin up and down, and *H*_SOC_ is the strength of SOC term) is fairly small due to crystal symmetries.^[^ ^30^ ^]^ In fact, such a tiny, undetectable opened gap would have little influence on the nodal line behavior. For example, ZrSiS is experimentally confirmed as a nodal line semimetal (the nodal line near Fermi level also exists in the *k_z_* = 0 plane without SOC), while it shows ≈20 meV opened gap in the DFT calculation when taking SOC into account.^[^ ^31^ ^]^ Compared with ZrSiS, the nodal line of YbMnSb_2_ has much smaller gap in the DFT calculation with SOC, indicating that YbMnSb_2_ also provides platform to investigate the nodal line behavior. In addition, a very small DOS is found near *E*_F_, which in turn indicates its Dirac‐like nature. Electrical transport properties would be contributed from both featured Dirac bands and the regular hole pocket, though the major charge carriers will be holes contributed by the regular band.

**Figure 2:**
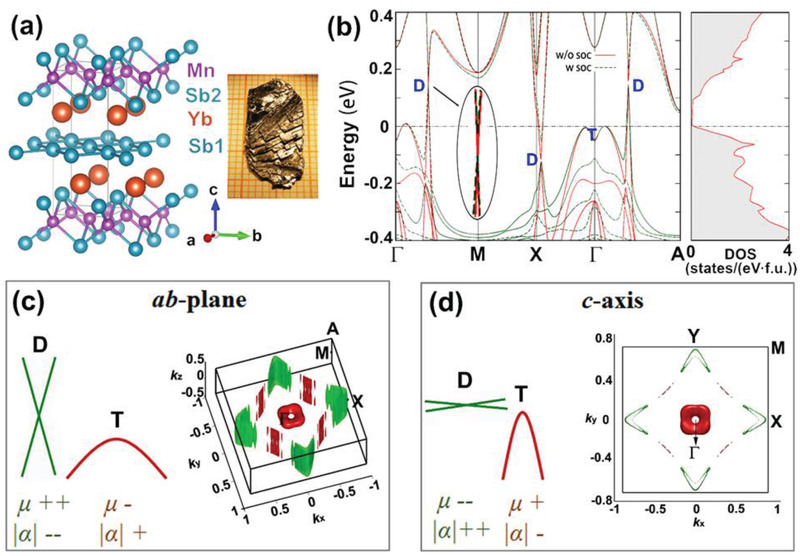
a) Crystal structure of YbMnSb_2_ and image of the as‐grown single crystal showing a clear layered structure. b) Calculated band structure and DOS, band structures with SOC (“w soc”) and without SOC (“w/o soc”) are both included. Here “D” presents the Dirac bands and “T” refers to traditional regular bands. A very small gap (<10 meV) in the Dirac bands can be introduced by SOC, and inset shows an enlargement of the Dirac band indicating the extremely small band gap due to SOC. c,d) Fermi surface illustration in the *ab*‐plane (c) and *c*‐axis direction (d); the dispersion of the Dirac bands is shown by the green solid lines.

Anisotropic dispersion of both the Dirac bands and the regular hole pocket along the *ab*‐plane and *c*‐axis in YbMnSb_2_ are shown in Figure 2c,d. The 2D Dirac bands are highly dispersive in the *k_x_*‐*k_y_* (*ab*‐plane ) direction but present almost no dispersion in the *k_z_* (*c*‐axis) direction. Differently, the regular hole pocket near Γ shows a “torus‐like” Fermi surface, indicating a larger *m*_b_^*^ in the *ab*‐plane direction while a smaller *m*_b_^*^ in the *c*‐axis direction. The differences in the band structure of YbMnSb_2_ along the two directions could give rise to a strong anisotropy in the transport properties. For analyzing the dominated bands to the electrical transport properties, we can first assume a scenario where only one type of band contributes to the electrical transport properties. Since the *m*_b_^*^ of the regular hole pocket is smaller in the *c*‐axis, lower resistivity is expected along the *c*‐axis if the regular band dominates the charge carrier transport. On the contrary, the 2D Dirac bands indicate a very high μ and thus lower resistivity along the *ab*‐plane, if the Dirac bands play a significant role in the electrical transport properties. Therefore, the fact how Dirac and regular bands contribute to the thermoelectric performance can be revealed by measuring the electrical transport properties along different directions.

The thermoelectric transport properties of YbMnSb_2_ along the *ab*‐plane and *c*‐axis are shown in **Figure** **3**. Noteworthy, the *ab*‐plane direction displays one order of magnitude lower resistivity than the *c*‐axis, suggesting that the highly dispersive 2D Dirac bands along the *ab*‐plane have a great effect on enhancing the electrical conduction. Hall measurement indicates that the mobility along the *ab*‐plane is more than ten times higher than that along the *c*‐axis (Figure S1, Supporting Information) at 300 K, while the charge carrier concentration along the *ab*‐plane and *c*‐axis is approximately the same around 9 × 10^19^ cm^−3^, giving a direct evidence of the highly dispersive Dirac bands enhanced electrical transport. The thermopower along the *ab*‐plane and *c*‐axis exhibits much less anisotropy. At 300 K, a value of ≈160 µV K^−1^ is obtained for both the *ab*‐plane and *c*‐axis, as the density of the states are mainly contributed by the regular hole pocket. The slightly higher Seebeck coefficient in the *c*‐axis may stem from the weak contribution of the flat Dirac bands. Notably, the large value of Seebeck coefficient confirms that the major charge carriers are holes, and the holes have a much larger concentration than the minor electrons. Consequently, the *ab*‐plane show much better electrical performance than the *c*‐axis, benefit mainly from the highly dispersive Dirac bands guaranteeing a high mobility, and partly also the anisotropic crystal structure. It is worth noting that the power factor along the *ab*‐plane direction is ten times higher than that of the *c*‐axis, reaching a value over 2.1 mW m^−1^ K^−2^ at 300 K (Figure 3c).

**Figure 3:**
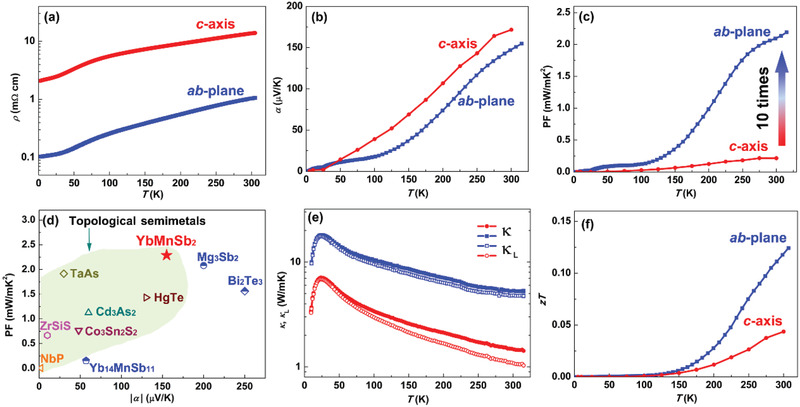
a–f) Temperature dependence of resistivity (a), Seebeck coefficient (b), power factor (c), total and lattice thermal conductivity (e), and *zT* values (f) for YbMnSb_2_ along the *ab*‐plane and *c*‐axis, respectively. d) Comparison of thermopower and power factor of several topological semimetals to high‐performance thermoelectric semiconductors at 300 K. To avoid the influence of other mechanisms, all the data are taken from single crystals without further doping or alloying.^[^ ^29^, ^30^, ^31^, ^32^, ^33^, ^34^, ^35^, ^36^, ^37^, ^38^, ^39^, ^40^ ^]^

To further demonstrate the potential of the unique band structure in YbMnSb_2_ for thermoelectrics, we compare the thermopower (|α|) and power factor of YbMnSb_2_ to other topological semimetals, including HgTe,^[^ ^32^ ^]^ TaAs,^[^ ^33^ ^]^ NbP,^[^ ^34^, ^35^ ^]^ Cd_3_As_2_,^[^ ^36^, ^37^ ^]^ ZrSiS,^[^ ^38^, ^39^ ^]^ Co_3_Sn_2_S_2_,^[^ ^40^ ^]^ as well as the good thermoelectric semiconductors, for example, Bi_2_Te_3_,^[^ ^41^ ^]^ Mg_3_Sb_2_,^[^ ^42^ ^]^ and Yb_14_MnSb_11_,^[^ ^43^ ^]^ as shown in Figure 3d. Generally, topological semimetals show a smaller |α| than that of thermoelectric semiconductors. Nevertheless, YbMnSb_2_ displays a surprisingly large |α|, which is one of the highest values among the present novel semimetals and comparable to that of good thermoelectric semiconductors. This is due to the unique configuration of the featured band and regular band, in which major charger carriers are much more than the minor charge carriers. Such a large thermopower along with low resistivity result in the high power factor over 2.1 mW m^−1^ K^−2^, which is even higher than the pure Bi_2_Te_3_^[^ ^41^ ^]^ and Mg_3_Sb_2_^[^ ^42^ ^]^ single crystals, based on which the optimized alloys show the best thermoelectric performance at around 300 K. Such an excellent power factor demonstrates the effectiveness of combing highly dispersive Dirac bands with regular bands (as schemed in Figure 1c) to significantly enhance the thermoelectric performance, as well as the potential of novel semimetals to be high performance thermoelectric materials.

An obvious anisotropy is also observed in thermal conductivity as well as the *zT* values, owing to the anisotropic crystal and band structure (Figure 3e,f). Interestingly, the lattice thermal conductivities of YbMnSb_2_ single crystal are quite low, which is only ≈1 W m^−1^ K^−1^ at 300 K along the *c*‐axis, being even lower than that of Bi_2_Te_3_. No significant bipolar thermal conductivity up to 300 K has been observed in both directions, indicating the ignorable contribution of minor charge carriers below room temperature due to the unique band configuration. Consequently, benefit from the greatly enhanced power factor, a maximum *zT* of ≈0.12 at 300 K is obtained along the *ab*‐plane, about three times higher than that of the *c*‐axis, which is a good value for a novel semimetal single crystal without any purposeful optimization.

Further strategies, for example, using multiple phonon scattering to decrease the thermal conductivity,^[^ ^44^ ^]^ optimizing the carrier concentration by chemical doping,^[^ ^45^ ^]^ as well as applying magnetic field^[^ ^46^, ^47^ ^]^ are promising to enhance the thermoelectric performance. Note that Zintl compounds are a big family composed of electropositive cations (typically, groups 1 and 2) and post‐transition metal or metalloid (i.e., groups 13–16), varying from strictly defined semiconducting Zintl phase to metallic alloys.^[^ ^48^ ^]^ YbMnSb_2_ is one member of the RMnPn_2_ Zintl family, where R is a rare‐earth or alkaline‐earth metal and Pn is a pnictide (P, As, Sb, or Bi). There are more RMnPn_2_ compounds, for example, YbMnBi_2_,^[^ ^49^ ^]^ SrMnSb_2_,^[^ ^50^ ^]^ SrMnBi_2_,^[^ ^51^ ^]^ CaMnBi_2_,^[^ ^51^ ^]^ and BaMnBi_2_,^[^ ^52^ ^]^ as well as some other 1‐1‐2 type compound have been experimentally proved to exhibit a 2D Dirac Fermi surface. All these compounds have similar layered structure as YbMnSb_2_, indicating intrinsically low lattice thermal conductivity. Therefore, the alloying of these compounds can reduce the thermal conductivity and probably also modulate the band structure, which would be advantageous for enhancing the thermoelectric performance.

In addition to the promising thermoelectric application, the Dirac nature and the intrinsically low thermal conductivity of YbMnSb_2_ are further explored by measuring the specific heat. As shown in **Figure** **4**a, a slight upturn of the specific heat is observed below 3 K under 0 T, which is probably due to the possible existence of magnetic impurities (which is almost unavoidable in Yb^2+^‐based compounds). In order to eliminate the magnetic impurities contribution, specific heat is measured under 9 T, which shows throughout decreasing trend with decreasing temperature. According to the Debye model, specific heat can be presented by the following equation when *T* << Θ:

(2) $$\begin{array}{c} C_{p}/T=\gamma +\frac{12\pi^{4}Nk_{B}}{5}{\left(\frac{1}{\Theta }\right)}^{3}T^{2} \end{array}$$

**Figure 4:**
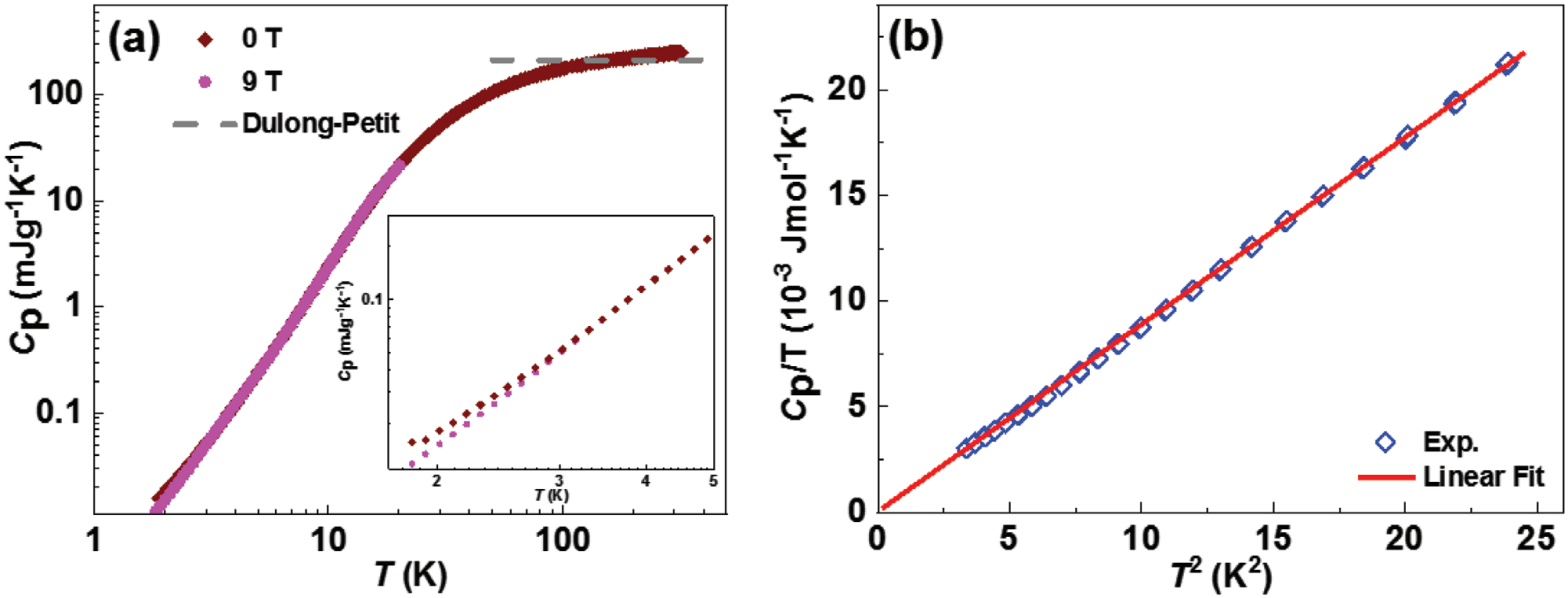
a) Temperature dependence of specific heat under 0 T and 9 T. b) Linear fitting of *T* ^2^ dependence of *C*_p_/*T* at very low temperatures below 5 K.

where *C*_p_ is specific heat with a unit of J mol^−1^ K^−1^, γ is the electronic specific heat, *N* is the number of atoms per mole (which is 4*N*_A_ for YbMnSb_2_, *N*_A_ is Avogadro constant), *k*_B_ is the Boltzmann constant, Θ is the Debye temperature.^[^ ^53^ ^]^ The specific heat under 9 T is used to obtain γ by linear fitting the *T* ^2^ dependence of *C*_p_/*T* at low temperatures (<5 K). As shown in Figure 4b, an extremely small γ value clarifies a near‐zero electronic specific heat, which proves the Dirac nature of YbMnSb_2_ at low temperatures.

From the slope of the *C*_p_/*T*–*T* ^2^ curve (Figure 4b), a Debye temperature of Θ = 204 K is obtained. Then the average sound velocity υ_s_ can be obtained by

(3) $$\begin{array}{c} k_{B}\Theta \;=\hbar {\left(\frac{6\pi^{2}}{V_{\text{atom}}}\right)}^{1/3}\upsilon_{s} \end{array}$$

where *ℏ* is the reduced Planck constant and *V*_atom_ is the average volume per atom.^[^ ^53^ ^]^ The resolved value of average sound velocity is 1990 m s^−1^, which is even lower than the state‐of‐the‐art Bi_2_Te_3_ thermoelectric material which has υ_s_ of 2147 and 2070 m s^−1^, along the *ab*‐plane and the *c*‐axis, respectively.^[^ ^54^ ^]^ This low value of sound velocity, which is generally related to the weak chemical bonding, is one of the reasons leading to the intrinsically low lattice thermal conductivity of YbMnSb_2_.

In conclusion, single crystals of the nodal line semimetal YbMnSb_2_ are tested to examine their unusual thermoelectric properties. For a material expected to be a semimetal, it exhibits a high Seebeck coefficient, low resistivity, and low thermal conductivity. The Dirac band likely plays a significant role in the material having a low resistivity. Simultaneously a regular band appears to participate in the transport properties leading to a transport behavior like that of a heavily doped, small band gap semiconductor. Hence a large Seebeck coefficient with no obvious bipolar thermal conductivity up to 300 K is observed. As a result, a room temperature *zT* value of 0.12 is achieved in the *ab*‐plane, with a 300% enhancement compared to the *c*‐axis where Dirac band is nearly non‐dispersive. The present work demonstrate that novel semimetals can be promising thermoelectric materials. Further *zT* enhancement is possible by reducing thermal conductivity, as well as alloying with other Zintl phases to engineer the band structure.

## Experimental Section

### Ab Initio Calculations

The structure was fully optimized based on the experimental data^[^ ^28^ ^]^ until the force on each atom was smaller than 0.01 eV Å^−1^. Spin‐polarized density functional calculations were performed by using Vienna ab initio Simulation Package.^[^ ^55^, ^56^ ^]^ The projector augmented wave method was used.^[^ ^57^ ^]^ The valence electrons for Yb are 5s, 5p, 4f, and 6s; for Mn are 3d and 4s; and for Sb are 5s and 5p. The PBE GGA functional^[^ ^58^ ^]^ was employed, and a Hubbard U correction was used for Yb 4f electrons with *U*_eff_ = 4eV (*U*_eff_ = 4eV on Yb 4f states reproduced the experimental photoemission results best).^[^ ^28^ ^]^ Note that the different *U*_eff_ values on Yb 4f states do not affect the conclusions significantly since the Yb 4f states are far away from the Fermi energy, and the bands near the Fermi energy are mainly *p_x_* and *p_y_* orbitals of Sb_1_ (Figure S7, Supporting Information). The plane‐wave function basis was used to expand the wave functions, and the energy cutoff was 400 eV. A 10 × 10 × 4 Monkhorst–Pack k mesh^[^ ^59^ ^]^ was generated for the self‐consistent calculations, a 16 × 16 × 10 one for density‐of‐state calculation, and a 50 × 50 × 50 one for Fermi surface calculations. An antiferromagnetic order was employed with the two Mn ions in the unit cell host opposite spin directions.

### Samples Preparation

YbMnSb_2_ single crystals were grown by a self‐flux method with Sb flux.^[^ ^60^ ^]^ Starting elements Yb (cut into small pieces, 99.99%), Mn (piece, 99.98%), and Sb (shot, 99.999%) were weighed and mixed with a molar ratio of Yb:Mn:Sb = 1:1:4. The mixtures were then transported in an alumina crucible, and then sealed in a quartz tube under partial argon pressure. The sealed tube was first heated up to 1050 °C in 3 days, kept for 48 h, and then slowly cooled down to 700 °C with a rate of 2 °C h^−1^. Single crystals were obtained after a centrifuging process.

### Samples Characterization

Single crystallinity and orientation of the as‐grown crystals were determined by white‐beam backscattering Laue X‐ray diffraction (XRD) method (Figure S2, Supporting Information). Phase purity was evaluated by powder XRD (Figure S3, Supporting Information). Composition and homogeneity were examined by scanning electron microscopy (Philips XL30) with Oxford energy‐dispersive X‐ray (EDX, Quantax, Bruker) apparatus (Figure S4, Supporting Information).

### Transport Properties Measurement

Resistivities (ρ) and Hall resistivities (ρ_H_) were measured simultaneously using a PPMS‐9T instrument (Quantum Design) in ACT mode via a standard four‐probe method. All measured data were field‐symmetrized and antisymmetrized to correct for the contact misalignment. Hall carrier concentration and mobility were obtained from *n*_H_ = 1/*eR*_H_, and μ_H_ = *R*_H_/ρ, where *R*_H_ is Hall coefficient. Thermal conductivities (κ) were measured in a home‐built high vacuum cryostat by adopting the standard four‐contact steady state method (Figure S5, Supporting Information). Seebeck coefficient (α) was measured using PPMS with one‐heater two‐thermometer configuration under high vacuum. The magnetization measurement (Figure S6, Supporting Information) was carried out using a Quantum Design MPMS3 instrument.

## Conflict of Interest

The authors declare no conflict of interest.

## Supporting information

Supporting Information

## References

[1] M. S. Dresselhaus , G. Chen , M. Y. Tang , R. Yang , H. Lee , D. Wang , Z. Ren , J.‐P. Fleurial , P. Gogna , Adv. Mater. 2007, 19, 1043.

[2] J. P. Heremans , V. Jovovic , E. S. Toberer , A. Saramat , K. Kurosaki , A. Charoenphakdee , S. Yamanaka , G. J. Snyder , Science 2008, 321, 554. 18653890 10.1126/science.1159725

[3] Y. Pei , X. Shi , A. LaLonde , H. Wang , L. Chen , G. J. Snyder , Nature 2011, 473, 66. 21544143 10.1038/nature09996

[4] Z. M. Gibbs , F. Ricci , G. Li , H. Zhu , K. Persson , G. Ceder , G. Hautier , A. Jain , G. J. Snyder , npj Comput. Mater. 2017, 3, 8.

[5] B. He , Y. Wang , M. Q. Arguilla , N. D. Cultrara , M. R. Scudder , J. E. Goldberger , W. Windl , J. P. Heremans , Nat. Mater. 2019, 18, 568. 30886402 10.1038/s41563-019-0309-4

[6] B. Skinner , L. Fu , Sci. Adv. 2018, 4, eaat2621. 29806031 10.1126/sciadv.aat2621 PMC5969823

[7] C. Fu , Y. Sun , C. Felser , APL Mater. 2020, 8, 040913.

[8] Y. L. Chen , J. G. Analytis , J.‐H. Chu , Z. K. Liu , S.‐K. Mo , X. L. Qi , H. J. Zhang , D. H. Lu , X. Dai , Z. Fang , S. C. Zhang , I. R. Fisher , Z. Hussain , Z.‐X. Shen , Science 2009, 325, 178. 19520912 10.1126/science.1173034

[9] B. Poudel , Q. Hao , Y. Ma , Y. Lan , A. Minnich , B. Yu , X. Yan , D. Wang , A. Muto , D. Vashaee , X. Chen , J. Liu , M. S. Dresselhaus , G. Chen , Z. Ren , Science 2008, 320, 634. 18356488 10.1126/science.1156446

[10] Y. Pan , Y. Qiu , I. Witting , L. Zhang , C. Fu , J.‐W. Li , Y. Huang , F.‐H. Sun , J. He , G. J. Snyder , C. Felser , J.‐F. Li , Energy Environ. Sci. 2019, 12, 624.

[11] H. Shi , D. Parker , M.‐H. Du , D. J. Singh , Phys. Rev. Appl. 2015, 3, 014004.

[12] J. P. Heremans , R. J. Cava , N. Samarth , Nat. Rev. Mater. 2017, 2, 17049.

[13] T. Fang , X. Li , C. Hu , Q. Zhang , J. Yang , W. Zhang , X. Zhao , D. J. Singh , T. Zhu , Adv. Funct. Mater. 2019, 29, 1906677.

[14] I. T. Witting , T. C. Chasapis , F. Ricci , M. Peters , N. A. Heinz , G. Hautier , G. J. Snyder , Adv. Electron. Mater. 2019, 5, 1800904.

[15] B. A. Bernevig , T. L. Hughes , S.‐C. Zhang , Science 2006, 314, 1757. 17170299 10.1126/science.1133734

[16] C.‐Z. Chang , J. Zhang , X. Feng , J. Shen , Z. Zhang , M. Guo , K. Li , Y. Ou , P. Wei , L.‐L. Wang , Z.‐Q. Ji , Y. Feng , S. Ji , X. Chen , J. Jia , X. Dai , Z. Fang , S.‐C. Zhang , K. He , Y. Wang , L. Lu , X.‐C. Ma , Q.‐K. Xue , Science 2013, 340, 167. 23493424 10.1126/science.1234414

[17] A. A. Burkov , Nat. Mater. 2016, 15, 1145. 27777403 10.1038/nmat4788

[18] B. Yan , C. Felser , Annu. Rev. Condens. Matter Phys. 2017, 8, 337.

[19] S. D. Kang , G. J. Snyder , Nat. Mater. 2017, 16, 252.27842076

[20] D. M. Rowe , Thermoelectrics Handbook: Macro to Nano, CRC Press, Boca Raton, FL, USA 2006.

[21] H.‐S. Kim , N. A. Heinz , Z. M. Gibbs , Y. Tang , S. D. Kang , G. J. Snyder , Mater. Today 2017, 20, 452.

[22] W. Liu , X. Tan , K. Yin , H. Liu , X. Tang , J. Shi , Q. Zhang , C. Uher , Phys. Rev. Lett. 2012, 108, 166601. 22680741 10.1103/PhysRevLett.108.166601

[23] M. Zhou , Z. M. Gibbs , H. Wang , Y. Han , C. Xin , L. Li , G. J. Snyder , Phys. Chem. Chem. Phys. 2014, 16, 20741. 25162449 10.1039/c4cp02091j

[24] G. Tan , F. Shi , S. Hao , H. Chi , L.‐D. Zhao , C. Uher , C. Wolverton , V. P. Dravid , M. G. Kanatzidis , J. Am. Chem. Soc. 2015, 137, 5100. 25856499 10.1021/jacs.5b00837

[25] Y. Tang , Z. M. Gibbs , L. A. Agapito , G. Li , H.‐S. Kim , M. B. Nardelli , S. Curtarolo , G. J. Snyder , Nat. Mater. 2015, 14, 1223. 26436339 10.1038/nmat4430

[26] a) N. F. Mott , E. A. Davis , Electronic Processes in Non‐Crystalline Materials, Clarendon, Oxford, UK 1971;

[27] Y. Xu , Z. Gan , S.‐C. Zhang , Phys. Rev. Lett. 2014, 112, 226801. 24949782 10.1103/PhysRevLett.112.226801

[28] R. Kealhofer , S. Jang , S. M. Griffin , C. John , K. A. Benavides , S. Doyle , T. Helm , P. J. W. Moll , J. B. Neaton , J. Y. Chan , J. D. Denlinger , J. G. Analytis , Phys. Rev. B 2018, 97, 045109.

[29] X. Wu , S. Qin , Y. Liang , C. Le , H. Fan , J. Hu , Phys. Rev. B 2015, 91, 081111(R).

[30] Z. Qiu , C. Le , Z. Liao , B. Xu , R. Yang , J. Hu , Y. Dai , X. Qiu , Phys. Rev. B 2019, 100, 125136.

[31] L. M. Schoop , M. N. Ali , C. Straßer , A. Topp , A. Varykhalov , D. Marchenko , V. Duppel , S. S. P. Parkin , B. V. Lotsch , C. R. Ast , Nat. Commun. 2016, 7, 11696. 27241624 10.1038/ncomms11696 PMC4895020

[32] M. Markov , X. Hu , H.‐C. Liu , N. Liu , S. J. Poon , K. Esfarjani , M. Zebarjadi , Sci. Rep. 2018, 8, 9876. 29959341 10.1038/s41598-018-28043-3 PMC6026171

[33] J. Xiang , S. Hu , M. Lv , J. Zhang , H. Zhao , G. Chen , W. Li , Z. Chen , P. Sun , J. Phys.: Condens. Matter 2017, 29, 485501. 29072578 10.1088/1361-648X/aa964b

[34] C. Shekhar , A. Nayak , Y. Sun , M. Schmidt , M. Nicklas , I. Leermakers , U. Zeitler , Y. Skourski , J. Wosnitza , Z. Liu , Y. Chen , W. Schnelle , H. Borrmann , Y. Grin , C. Felser , B. Yan , Nat. Phys. 2015, 11, 645.

[35] S. J. Watzman , T. M. McCormick , C. Shekhar , S.‐C. Wu , Y. Sun , A. Prakash , C. Felser , N. Trivedi , J. P. Heremans , Phys. Rev. B 2017, 97, 161404.

[36] H. Wang , X. Luo , W. Chen , N. Wang , B. Lei , F. Meng , C. Shang , L. Ma , T. Wu , X. Dai , Z. Wang , X. Chen , Sci. Bull. 2018, 63, 411. 10.1016/j.scib.2018.03.010 36658935

[37] H. Wang , X. Luo , K. Peng , Z. Sun , M. Shi , D. Ma , N. Wang , T. Wu , J. Ying , Z. Wang , X. Chen , Adv. Funct. Mater. 2019, 29, 1902437.

[38] M. Matusiak , J. Cooper , D. Kaczorowski , Nat. Commun. 2017, 8, 15219. 28537261 10.1038/ncomms15219 PMC5529674

[39] R. Singha , A. K. Pariari , B. Satpati , P. Mandal , Proc. Natl. Acad. Sci. USA 2017, 114, 2468. 28223488 10.1073/pnas.1618004114 PMC5347568

[40] P. Mangelis , P. Vaqueiro , J. C. Jumas , I. da Silva , R. I. Smith , A. V. Powell , J. Solid State Chem. 2017, 251, 204.

[41] H. W. Jeon , H. P. Ha , D. B. Hyun , J. D. Shim , J. Phys. Chem. Solids 1991, 52, 579.

[42] K. Imasato , C. Fu , Y. Pan , M. Wood , J. J. Kuo , C. Felser , G. J. Snyder , Adv. Mater. 2020, 32, 1908218. 10.1002/adma.201908218 32115799

[43] J. F. Rauscher , C. A. Cox , T. Yi , C. M. Beavers , P. Klavins , E. S. Toberer , G. J. Snyder , S. M. Kauzlarich , Dalton Trans. 2010, 39, 1055. 20066191 10.1039/b920250a

[44] K. Biswas , J. He , I. D. Blum , C. I. Wu , T. P. Hogan , D. N. Seidman , V. P. Dravid , M. G. Kanatzidis , Nature 2012, 489, 414. 22996556 10.1038/nature11439

[45] G. J. Snyder , E. S. Toberer , Nat. Mater. 2008, 7, 105. 18219332 10.1038/nmat2090

[46] J. Xiang , S. Hu , M. Lyu , W. Zhu , C. Ma , Z. Chen , F. Steglich , G. Chen , P. Sun , Sci. China: Phys., Mech. Astron. 2020, 63, 237011.

[47] C. Fu , S. N. Guin , S. J. Watzman , G. Li , E. Liu , N. Kumar , V. Süβ , W. Schnelle , G. Auffermann , C. Shekhar , Y. Sun , J. Gooth , C. Felser , Energy Environ. Sci. 2018, 11, 2813.

[48] E. S. Toberer , A. F. May , G. J. Snyder , Chem. Mater. 2010, 22, 624.

[49] S. Borisenko , D. Evtushinsky , Q. Gibson , A. Yaresko , K. Koepernik , T. Kim , M. Ali , J. van den Brink , M. Hoesch , A. Fedorov , E. Haubold , Y. Kushnirenko , I. Soldatov , R. Schäfer , R. J. Cava , Nat. Commun. 2019, 10, 3424. 31366883 10.1038/s41467-019-11393-5 PMC6668437

[50] J. Y. Liu , J. Hu , Q. Zhang , D. Graf , H. B. Cao , S. M. A. Radmanesh , D. J. Adams , Y. L. Zhu , G. F. Cheng , X. Liu , W. A. Phelan , J. Wei , M. Jaime , F. Balakirev , D. A. Tennant , J. F. DiTusa , I. Chiorescu , L. Spinu , Z. Q. Mao , Nat. Mater. 2017, 16, 905. 28740190 10.1038/nmat4953

[51] A. Zhang , C. Liu , C. Yi , G. Zhao , T.‐l. Xia , J. Ji , Y. Shi , R. Yu , X. Wang , C. Chen , Q. Zhang , Nat. Commun. 2016, 7, 13833. 27982036 10.1038/ncomms13833 PMC5172363

[52] L. Li , K. Wang , D. Graf , L. Wang , A. Wang , C. Petrovic , Phys. Rev. B 2016, 93, 115141.

[53] N. W. Ashcroft , N. D. Mermin , Solid State Physics, Holt, Rinehart and Winston, New York 1976.

[54] F. Yang , T. Ikeda , G. J. Snyder , C. Dames , J. Appl. Phys. 2010, 108, 034310.

[55] G. Kresse , J. Hafner , Phys. Rev. B 1993, 48, 13115. 10.1103/physrevb.48.13115 10007687

[56] G. Kresse , J. Furthmüller , Phys. Rev. B 1996, 54, 11169. 10.1103/physrevb.54.11169 9984901

[57] P. E. Blöchl , Phys. Rev. B 1994, 50, 17953. 10.1103/physrevb.50.17953 9976227

[58] J. P. Perdew , K. Burke , M. Ernzerhof , Phys. Rev. Lett. 1996, 77, 3865. 10062328 10.1103/PhysRevLett.77.3865

[59] H. J. Monkhorst , J. D. Pack , Phys. Rev. B 1976, 13, 5188.

[60] Y.‐Y. Wang , S. Xu , L.‐L. Sun , T.‐L. Xia , Phys. Rev. Mater. 2018, 2, 021201(R).

