# Supplementary material for: Thermoelectric Properties of Novel Semimetals: A Case Study of YbMnSb2
Source: Adv Mater. 2020 Dec 9;33(7):2003168. doi: 10.1002/adma.202003168 (PMC12121705; doi:10.1002/adma.202003168)
Supplement: Supplementary file 1 — Supporting Information [file ADMA-33-2003168-s001.pdf]

© 2020 Wiley-VCH GmbH

ADVANCED MATERIALS

Supporting Information

for *Adv. Mater.*, DOI: 10.1002/adma.202003168

Thermoelectric Properties of Novel Semimetals: A Case Study of $YbMnSb_2$

Yu Pan, Feng-Ren Fan, Xiaochen Hong, Bin He, Congcong Le, Walter Schnelle, Yangkun He, Kazuki Imasato, Horst Borrmann, Christian Hess, Bernd Büchner, Yan Sun, Chenguang Fu,* G. Jeffrey Snyder, and Claudia Felser**

© 2020 Wiley-VCH GmbH

Supporting Information

Thermoelectric properties of novel semimetals: a case study of $YbMnSb_2$

Author(s), and Corresponding Author(s)* Yu Pan[a],*, Feng-Ren Fan[a], Xiaochen Hong[b], Bin He[a], Congcong Le[a], Walter Schnelle[a], Yangkun He[a], Kazuki Imasato[c], Horst Borrmann[a], Christian Hess[b], Bernd Büchner[b,d], Yan Sun[a], Chenguang Fu[a],*, G. Jeffrey Snyder[c], Claudia Felser[a],*

Hall measurements along two directions

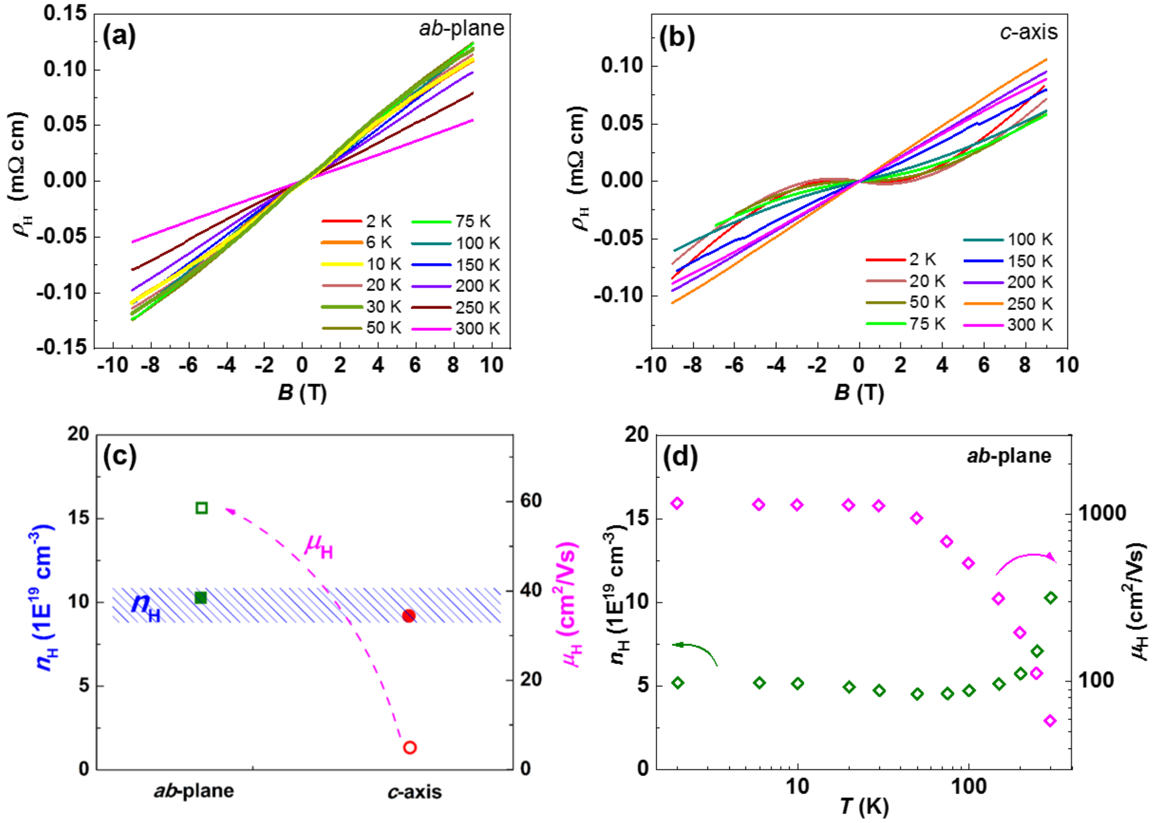

Figure S1. Magnetic field dependence of Hall resistivity along (a) *ab*-plane, (b) *c*-axis, respectively. (c) Hall charge carrier concentration and Hall charge carrier mobility at 300 K in

two directions. (d) Temperature dependence of Hall charge carrier concentration and Hall charge carrier mobility of *ab*-plane direction.

Hall resistivity ρ_H has some difference along *ab*-plane and *c*-axis (**Figure S1**(a) and (b)). At low temperatures below 100 K, ρ_H along *c*-axis shows a nonlinear magnetic field dependence, indicating a two types of charge carrier conduction. With increasing temperature, the Fermi level moves deeper into the trivial valence band, leading to a linear Hall resistivity dependent on magnetic field. This indicates that the major charge carriers are holes above 100 K.

The slope of magnetic field dependence of Hall resistivity is the Hall coefficient R_H, from which we can calculate the Hall charge carrier concentration by $n_H=1/eR_H$, and Hall charge carrier mobility $\mu_H=R_H/\rho$.[1] As shown in **Figure S1**(c), at 300 K the Hall charge carrier concentration values show negligible difference between the *ab*-plane and *c*-axis directions, while the mobility are more than ten times higher in the *ab*-plane than *c*-axis. **Figure S1**(d) further exhibits the temperature dependence of the Hall charge carrier concentration and mobility, where a surprisingly high mobility over 1200 cm^2/Vs is found at 2 K. Such a high mobility again demonstrates the potential of $YbMnSb_2$ as a good thermoelectric material.

<u>Single crystal quality</u>

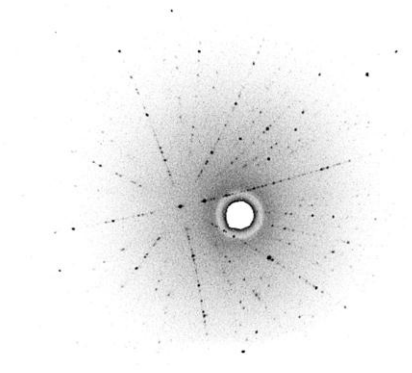

Figure S2. Laue pattern of $YbMnSb_2$ single crystal.

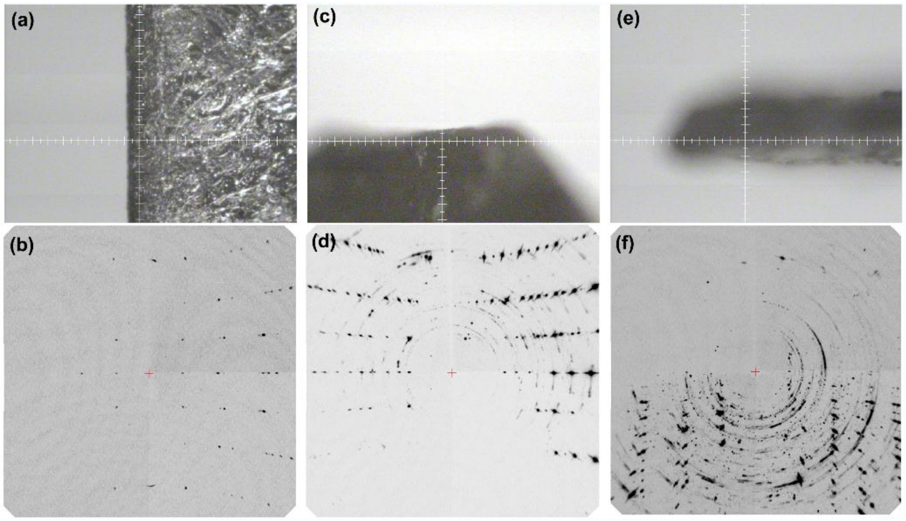

Figure S3. Orientation of the single crystal with the respective X-ray diffraction axial oscillation patterns. (a) and (b) are reflecting oscillations about *a*-axis, (c) and (d) about *b*-axis and (e) and (f) about *c*-axis.

Both Laue and single crystal XRD diffraction patterns (**Figure S2** and **Figure S3**) show distinct spots, demonstrating the good quality of the crystal. Reflection broadening in **Figure S3**(f) is due to slight surface distortion.

Composition analysis

The EDX results in **Figure S4** indicate the composition ratio of Yb:Mn:Sb as 24.3:24.3:51.4, which agrees well with the stoichiometric composition. The homogenous elemental mapping indicates uniform dispersion of the elements.

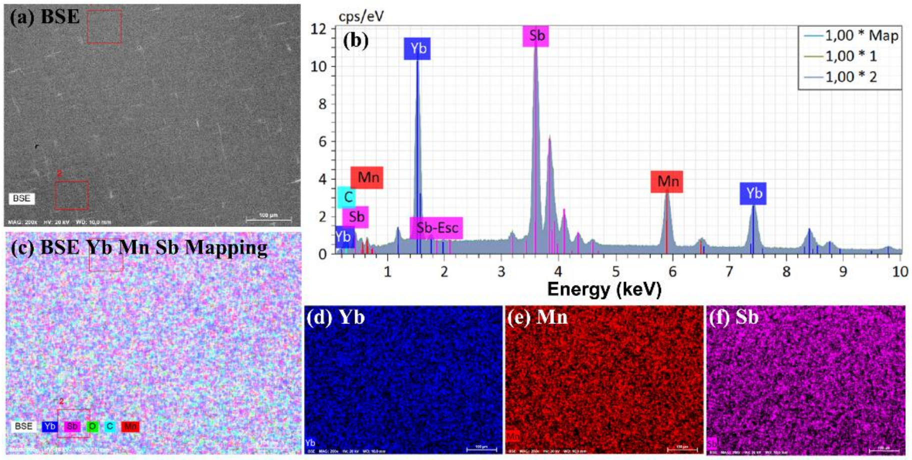

Fig. S4 (a) Backscattered electrons (BSE) image, (b) Energy-dispersive X-ray spectroscopy (EDX), (c) Yb, Mn, and Sb mapping, (d) Yb mapping, (e) Mn mapping, (f) Sb mapping of the single crystal.

Thermal conductivity measurement

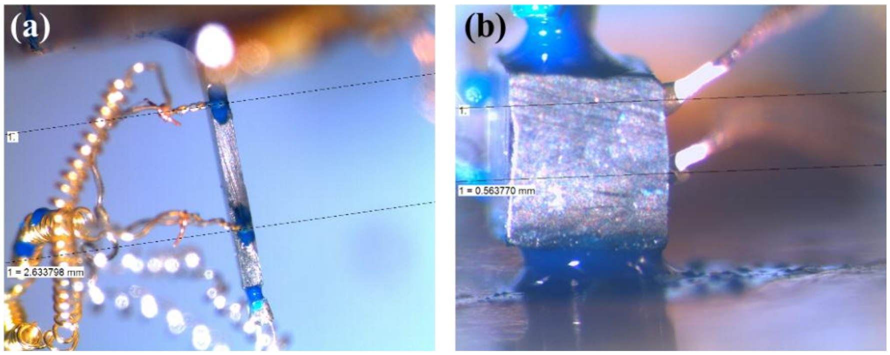

Figure S5. Sample setup for thermal conductivity measurement. (a) *ab*-plane, (b) *c*-axis

The sample was glued on the thermal bath with DeltaBond™ 152 adhesive, which is electrically insulating. The temperature gradient was applied by a chip resistor glued on the other end of the sample. The temperature gradient along the sample was measured with differential AuFe/Chromel thermocouples.

Magnetism measurement

The obvious magnetization upturn in the B//c direction, along with the magnetization hysteresis loops indicate an antiferromagnetic nature of $YbMnSb_2$ with magnetic moment along *c*-axis, as shown in **Figure S6**.

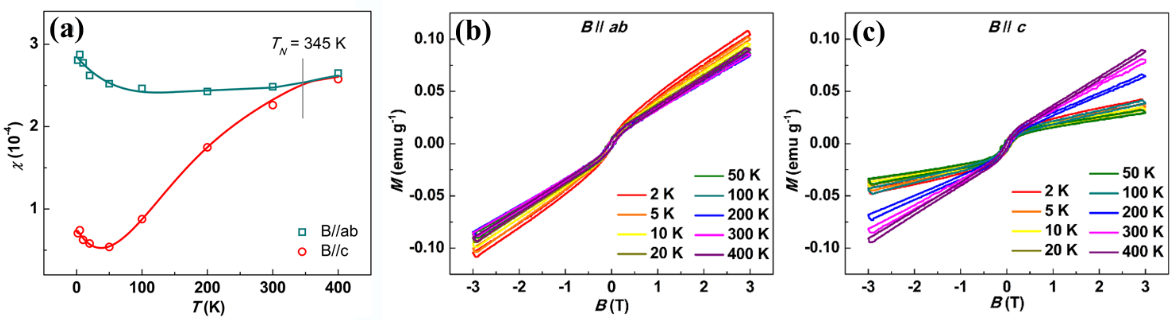

Figure S6. (a) Magnetization at various temperatures, and temperature dependence of magnetization with the magnetic field, (b) *ab*-plane and (c) *c*-axis direction.

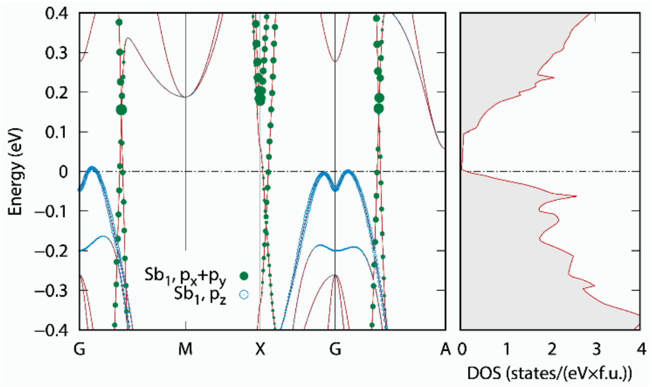

Figure S7. Band structure and DOS of $YbMnSb_2$ denoting the band characteristics near the Fermi level. The bands near the Fermi level are mainly are p_x and p_y orbitals of Sb_1.

References

[1]. N. W. Ashcroft, N. D. Mermin, *Solid state physics*, Holt, Rinehart and Winston, 1976.
